# Dynamic changes in OTULIN and progranulin levels in experimental myocardial infarction and cardiac remodeling

**DOI:** 10.1007/s00418-026-02506-5

**Published:** 2026-06-30

**Authors:** Sercan Kaya, İdris Doğan

**Affiliations:** 1 Medical Laboratory Program, Vocational Higher School of Healthcare Studies, Health Services Vocational School, Batman University, Main Campus, Room 212, Kültür Neighborhood, Batman, Turkey; 2 Physiotherapy Program, Vocational Higher School of Healthcare Studies, Batman University, Batman, Turkey

**Keywords:** Cardiac remodeling, Myocardial infarction, OTULIN, Progranulin

## Abstract

Oxidative stress and inflammation, in particular, are crucial factors in the pathogenesis of myocardial infarction (MI). Isoproterenol (ISO) is frequently preferred for creating experimental MI models because it reflects the pathophysiology of MI in humans. The purpose of this study was to identify potential alterations in OTULIN and progranulin (PRGN) levels at various stages during and following cardiac remodeling in experimental MI caused by ISO. The study consisted of control groups and ISO groups (ISO-6-h, ISO-24-h, ISO-3-day, and ISO-7-day) representing important time points in experimental MI and subsequent cardiac remodeling. The experimental MI model induced by ISO was validated by increased serum cardiac markers and histopathological changes in the heart tissue. Furthermore, it was determined that oxidative stress and inflammation, important factors in MI pathophysiology, emerged in the heart tissue after ISO administration. However, in the ISO-7-day group, a significant reduction in oxidative stress and inflammation was observed along with cardiac remodeling. In the experimental MI model, a time-dependent decrease in cardiac OTULIN levels was observed, while conversely, an increase in cardiac PRGN levels was detected. These time-dependent dynamic changes in cardiac OTULIN and PRGN suggest that both may play a role in processes related to MI and cardiac remodeling. In conclusion, endogenous OTULIN and PRGN may be associated with oxidative stress and inflammation in cardiac remodeling during and after experimental MI. This reveals the potential of OTULIN and PRGN as cardiac biomarkers in MI prognosis and suggests they could also be molecular targets for therapeutic strategies.

## Introduction

Coronary artery disease, one of the main causes of increasing mortality and morbidity rates worldwide, is most commonly known as myocardial infarction (MI) (Rong et al. 2023). Often referred to as a heart attack, MI is a pathophysiological condition resulting from insufficient blood flow to a portion of the myocardium, leading to impaired myocardial function (Singh and Jat 2021). One catecholamine that can be purchased synthetically is isoproterenol (ISO). ISO is used as a standard MI triggering agent in experimental research investigating the protective and therapeutic effects of MI (Govindasami et al. 2020; Kaya and Yalcin 2026). Among the proposed mechanisms in the ISO-induced experimental MI model, the imbalance between oxygen supply and demand of cardiomyocytes is particularly noteworthy (Ahmad et al. 2022). Therefore, ISO is frequently preferred in research because it reflects most of the metabolic and morphological abnormalities observed in human MI in experimental models (Kaya and Yalçın 2023; Saifullah et al. 2025).

Following MI, excessive inflammation and impaired cardiac healing can lead to heart failure. The cardiac repair process begins immediately after MI and can continue for months. This process is divided into three phases that overlap, but each have different functions and outcomes: the inflammatory phase, the proliferative phase, and the maturation phase (Zhang et al. 2023). The inflammatory phase lasts up to 4 days after MI in humans and up to 3 days in experimental rodent models. Beginning immediately after MI, this phase is the immune system’s inflammatory response to repair damaged heart tissue. The proliferative phase, which starts with the resolution of the post-MI inflammatory phase, is distinguished by the formation of early granulation tissue. This phase, during which anti-inflammatory and reparative cytokines are released, lasts up to day 14 after MI in humans and up to day 7 in experimental rodent models. The last stage of cardiac repair, known as the maturation phase, can take months to complete. During this time, low-level inflammation may cause cardiomyocyte death, inhibit cardiac contractility, and result in further extracellular matrix remodeling (Prabhu and Frangogiannis 2016). During these three phases, dysregulated inflammation can cause structural and functional changes in the ventricle, reducing cardiac output. This process, known as adverse cardiac remodeling, can ultimately lead to heart failure (Reed et al. 2017).

OTULIN, a deubiquitination enzyme, has various biological functions in the kidney, liver, testis, heart disease, and cancer. Most notably, it has an anti-inflammatory effect (Kaya and Yalcin 2024; Yalcin et al. 2024a, 2024b; Wang et al. 2024). For example, OTULIN deficiency in mouse macrophages leads to systemic inflammation (Hoste et al. 2021). OTULIN plays a crucial role in the regulation of inflammation in both humans and mice. Phenotypic changes due to OTULIN deficiency are primarily closely related to TNF (Damgaard et al. 2016). However, NF-κB activation’s linear ubiquitin chain assembly complex (LUBAC) is linked to immunity and inflammation. An important part of the inflammatory response mediated by the NF-κB pathway is M1-coupled linear inflammation. The linear ubiquitin chain’s poly-ubiquitination signal is controlled by OTULIN (Yalcin and Kaya 2026). Furthermore, M1-polyUb is selectively hydrolysed by OTULIN (Keusekotten et al. 2013). Furthermore, progranulin (PRGN), expressed in immune cells such as macrophages and neutrophils, has many functions associated with wound healing and immune modulation (Schmitz et al. 2020). Studies have reported that PRGN exhibits a protective effect against ischemic pathology in the kidney and brain (Egashira et al. 2013; Zhou et al. 2015). Additionally, PRGN treatment has been reported to provide protection against post-MI cardiac damage in various experimental models (rabbits and mice) (Sasaki et al. 2020). However, the role of endogenous PRGN and OTULIN in cardiac remodeling during and after MI, and their relationship with other cytokines and enzymes involved in the process, remains unclear.

Therefore, the aim of this study is to determine the possible changes in endogenous PRGN and OTULIN levels during different phases of cardiac remodeling during and after ISO-induced MI, based on oxidative stress and inflammation.

## Materials and methods

### Experimental design

The study commenced with the approval of the Firat University Animal Experiments Ethics Committee, dated 3 February 2026 and numbered 44167. All procedures during the experiment were carried out in accordance with the Animal Research: Reporting of In Vivo Experiments (ARRIVE) guidelines. Thirty Sprague–Dawley rats (8–10 weeks old, female, 200 ± 20 g) were used in the study. The rats were housed under optimal conditions (22–25 °C, 12-h light cycle, etc.) and fed with standard rat food (in pellet form) and water ad libitum. The rats were randomly divided into five equal groups (*n* = 6).

The control (*n* = 6) group, which did not undergo any procedures, was decapitated along with the ISO-7-day group.

In the ISO-6-h group (*n* = 6), rats were decapitated 6 h after myocardial infarction was induced by a single subcutaneous dose of 200 mg/kg ISO (I5627, isoproterenol hydrochloride, Sigma-Aldrich, St. Louis, MO, USA) dissolved in saline.

In the ISO-24-h group (*n* = 6); rats were decapitated 24 h after MI was induced using the same method.

In the ISO-3-day group (*n* = 6), using the same method, MI was induced in rats, and then the rats were decapitated 3 days later.

In the ISO-7-day group (*n* = 6), using in the same method, MI was induced in rats, and then the rats were decapitated 7 days later.

The number of rats in the experimental groups was determined by Gpower analysis. No adverse events or rat deaths were reported during the experiment. After all experimental procedures were completed, rats were decapitated at designated time points under anesthesia (xylazine 10 mg/kg and ketamine 75 mg/kg), and intracardiac blood samples were taken. Then, heart tissues were rapidly removed and used in analyses along with blood serum samples.

### Biochemical assessments

Serum levels of commonly used cardiac markers in clinical practice, troponin I (Tn-I; E0305Ra) and creatine phosphokinase-MB (CK-MB; E0311Ra), were determined using enzyme-linked immunosorbent assay (ELISA). Additionally, a portion of the obtained heart tissue was homogenized in 10% phosphate buffer solution (+4 °C, 10 min, 5000 × *g*). The supernatants of the heart tissue were collected after homogenization by centrifugation. Quantitative measurements of PRGN (E1222Ra) and OTULIN (E3387Ra) levels in heart tissue were determined using rat-specific ELISA kits, following the manufacturer’s instructions. In addition, oxidative stress parameters (malondialdehyde [MDA; E0156Ra] and 4-HNE [EA0066Ra]) and antioxidant enzyme levels (glutathione peroxidase [GPx; E1242Ra] and catalase [CAT; E0869Ra]) were determined in cardiac tissues using the ELISA method. Finally, proinflammatory marker levels (TNF-α [E0764Ra] and NF-κB [E0287Ra]) were determined in cardiac tissue homogenate using the ELISA method. All ELISA kits used in the study were obtained from Bioassay Technology Laboratory (BT Lab., Shanghai Korain Bio., Shanghai, China).

### Histopathological examinations

After the completion of the experimental procedures, some of the heart tissue samples were placed in a 10% formalin solution for histopathological evaluation. Following formalin fixation, they were processed through routine histology laboratory tissue processing lines and placed on paraffin blocks. The 5 µm thick sections were taken from the heart tissue in the paraffin blocks and subjected to hematoxylin–eosin staining for histopathological examination. Light microscopy (×20 objective, Air, NA 0.50, Leica Microsystems, DM2500 LED and MC170 HD, Wetzlar, Germany) was used for histopathological examinations and imaging. In the histopathological evaluation, ten randomly selected non-overlapping areas were examined at ×20 magnification in the heart tissue sections prepared separately for each rat and scored (according to the presence of the histopathological criterion: 0 = absent, 1 = minor, 2 = moderate, 3 = major). A Histopathological Evaluation Score (HES) was calculated for each preparation by scoring four separate criteria, with a total maximum score of 12 points. The histopathological criteria were mononuclear cell infiltration, myofibril loss, erythrocyte extravasation, and intracytoplasmic vacuolization.

### Immunohistochemical examinations

The 5 µm-thick sections were taken from the prepared paraffin blocks of heart tissue and placed on polylysin-coated slides. After deparaffinization and clearing, the heart tissue sections were passed through decreasing series of alcohols. Immunohistochemical (IHC) staining was performed on the heart tissues using the avidin–biotin–peroxidase complex method and the enzyme substrate AEC chromogen for PRG (Affinity, DF7997, Camarillo, CA, USA), Interleukin (IL)−1β (Santa Cruz, sc-1251, Dallas, Texas, USA), OTULIN (Boster, A07938-1, Pleasanton, CA, USA), TNF-α (Affinity, AF7014, Camarillo, CA, USA) and NF-κB (Affinity, AF5006, Camarillo, CA, USA). In IHC assessment, according to the previously described method (Kaya et al. 2023), the extent of immunostaining was calculated by multiplying it by the intensity of immunostaining. To ensure the reproducibility of the experiments, the catalog numbers of all antibodies used in the study and the full addresses of the suppliers were reported. Furthermore, the primary antibodies used (IL-1β, TNF-α, NF-κB, OTULIN, and PRGN) were validated for specificity by the manufacturers and confirmed with positive/negative controls under the Antibody Validation Policy in the Springer Publication Guidelines.

### Statistical analyses

Statistical analyses were performed using SPSS 22.0 software. The Shapiro–Wilk test was used to check the normality of the obtained data. For biochemical ELISA results and HES values showing normal distribution, the one-way analysis of variance (ANOVA) post hoc Tukey test was used. For IHC results that did not show normal distribution, the Kruskal–Wallis test and the Mann–Whitney *U* test were used for pairwise comparisons. A *p*-value < 0.05 was considered statistically significant. GraphPad Prism 8.4 software was used for the visual graphical presentation of the data.

## Results

### Serum cardiac marker levels at different time points after ISO application

**Fig. 1:**
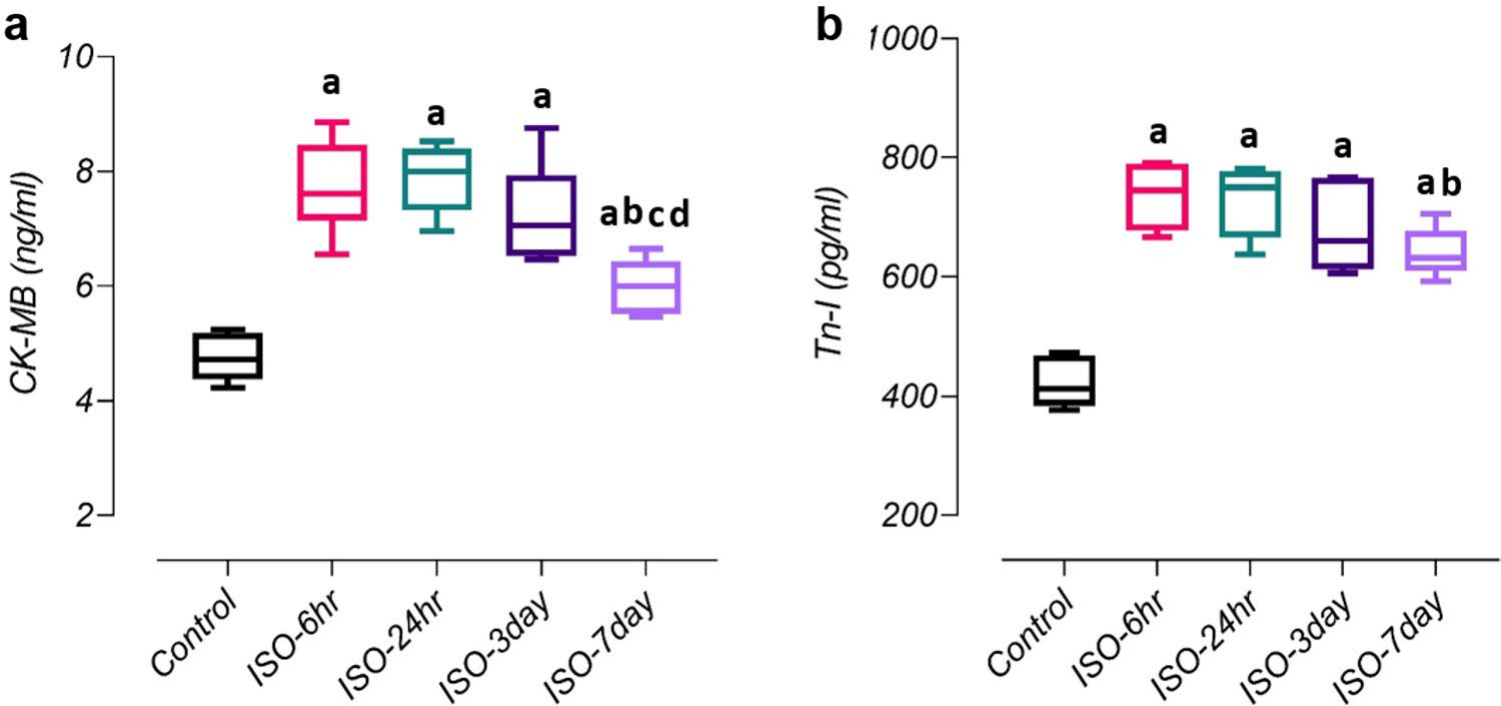
Circulating cardiac marker levels after ISO administration. Serum CK-MB and Tn-I levels increased in all ISO-treated groups compared with the control group. CK-MB levels were higher in the ISO-7-day group compared with the control group, while they were lower compared with other ISO groups. Tn-I levels were higher in the ISO-7-day group compared with the control group, while they were lower compared with the ISO-6-h group. **a** Cardiac creatine kinase MB (CK-MB) level graph. **b** Cardiac troponin I (Tn-I) level graph. ^a^Compared with the control group. ^b^Compared with the ISO-6-h group. ^c^Compared with the ISO-24-h group. ^d^Compared with the ISO-3-day group. *p* < 0.05. *ISO* isoproterenol

Serum CK-MB and Tn-I levels increased at 6 h, 24 h, 3 days, and 7 days after ISO administration compared with the control group (*p* < 0.05). However, serum CK-MB and Tn-I levels were similar in the ISO-24-h and ISO-3-day groups compared with the ISO-6-h group (*p* > 0.05). Serum CK-MB and Tn-I levels were decreased in the ISO-7-day group compared with the ISO-6-h group (*p* < 0.05). In the ISO-24-h group, serum CK-MB and Tn-I levels were similar to the ISO-3-day group (*p* > 0.05). However, when compared with the ISO-24-h group, serum CK-MB levels decreased in the ISO-7-day group (*p* < 0.05), while serum Tn-I levels were similar (*p* > 0.05). Similarly, serum CK-MB levels decreased in the ISO-7-day group compared with the ISO-3-day group (*p* < 0.05), while there was no difference in serum Tn-I levels (*p* > 0.05) (Fig. 1).

### Oxidative stress parameters and antioxidant enzyme levels in cardiac tissue after ISO application

**Fig. 2:**
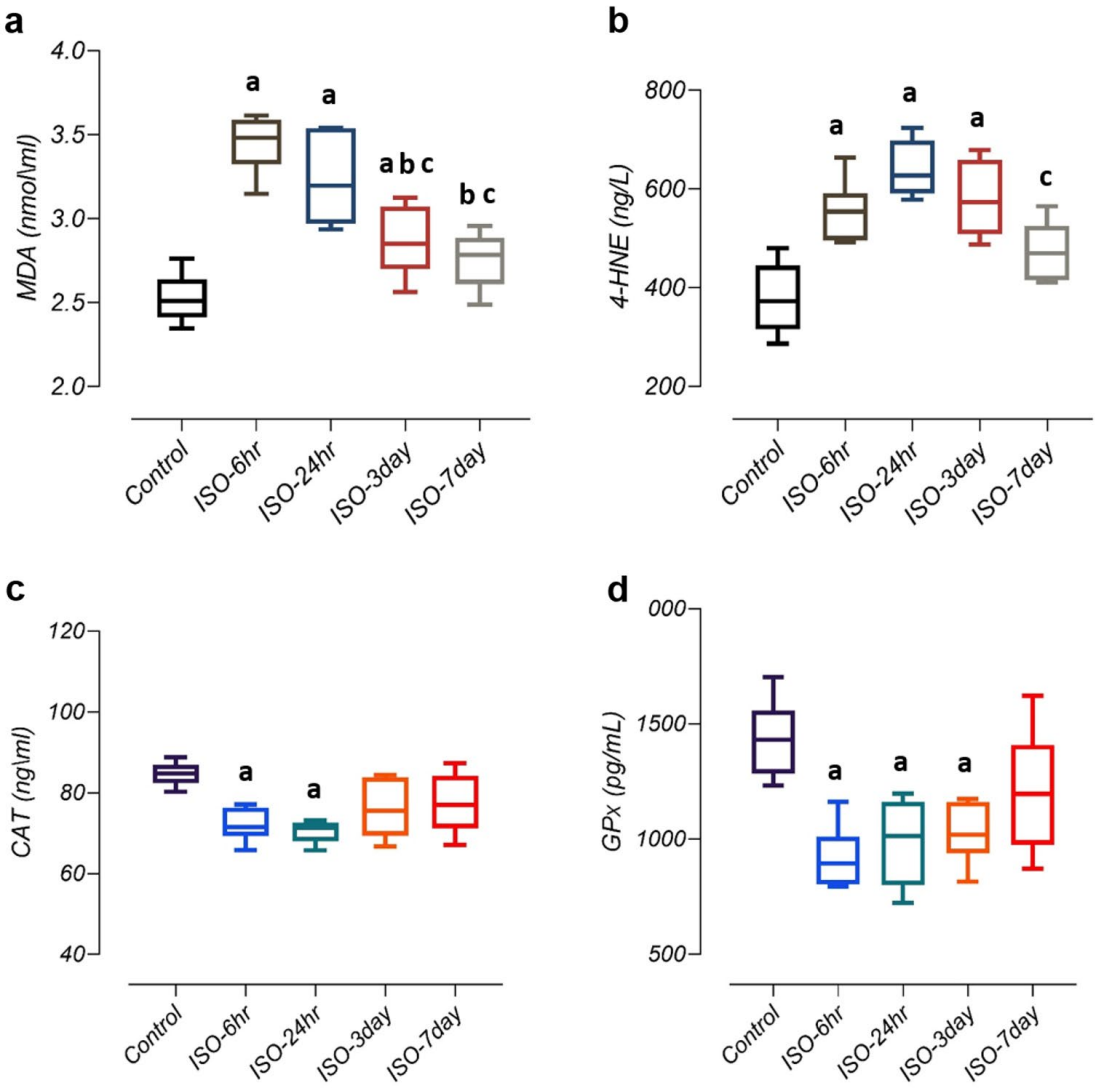
Oxidative stress parameters and antioxidant enzyme levels in cardiac tissue at different time points during and after the experimental MI process induced by ISO. In cardiac tissue, MDA and 4-HNE levels increased at 6 h, 24 h, and 3 days after ISO administration compared with the control group. However, on the 7th day after ISO administration, MDA and 4-HNE levels were not different from the control group. Furthermore, CAT levels decreased at 6 and 24 h after ISO administration, while GPx levels decreased at 6 h, 24 h, and 3 days compared with the control group. **a** cardiac tissue malondialdehyde (MDA) level. **b** cardiac tissue 4-hydroxynonenal (4-HNE) level. **c** cardiac tissue Catalase (CAT) level. **d** cardiac tissue Glutathione Peroxidase (GPx) level. ^a^Compared with the control group, ^b^Compared with the ISO-6-h group, ^c^Compared with the ISO-24-h group, *p* < 0.05

In cardiac tissue, MDA levels increased at 6 h, 24 h, and 3 days after ISO administration compared with the control group (*p* < 0.05). However, MDA levels decreased in the ISO-3-day and ISO-7-day groups compared with the ISO-6-h and ISO-24-h groups (*p* < 0.05). Similarly, 4-HNE levels increased in cardiac tissue at 6 h, 24 h, and 3 days after ISO administration compared with the control group (*p* < 0.05). However, a decrease in 4-HNE levels was observed in the ISO-7-day group compared with the ISO-24-h group (*p* < 0.05). Furthermore, CAT levels decreased at 6 and 24 h after ISO application compared with the control group (*p* < 0.05). However, CAT levels in the ISO-3-day and ISO-7-day groups were not different from the control group (*p* > 0.05). Similarly, GPx levels decreased at 6 h, 24 h, and 3 days after ISO application compared with the control group (*p* < 0.05). However, GPx levels in the ISO-7-day group were not different when compared with the control group (*p* > 0.05) (Fig. 2).

### Effect of ISO application on cardiac tissue histopathology at different time points

**Fig. 3:**
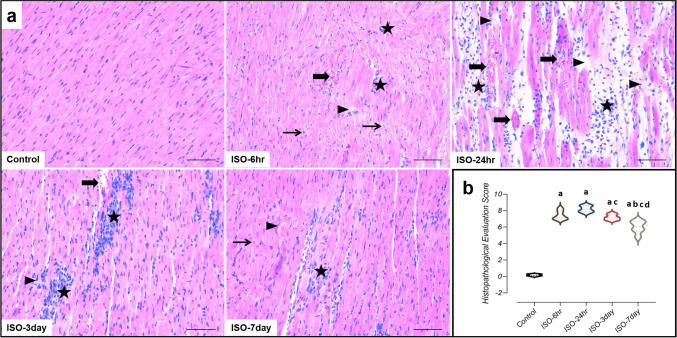
Cardiac histopathology at different time points after ISO application. Cardiac tissue in the control group exhibited normal histological structure. Widespread histopathological changes were observed 6 and 24 h after ISO administration, while a time-dependent decrease in histopathological changes was detected on days 3 and 7. **a** Hematoxylin and eosin staining. Scale bar 100 µm. Star: Mononuclear cell infiltration, Thick arrow: Erythrocyte extravasation, Arrowhead: Myofibril loss, Thin arrow: Intracytoplasmic vacuolization. **b** Histopathological evaluation score graph. ^a^Compared with the control group, ^b^Compared with the ISO-6-h group, ^c^Compared with the ISO-24-h group, ^d^Compared with the ISO-3-day group, *p* < 0.05. *ISO* isoproterenol

Cardiac tissue in the control group showed a normal histological structure. However, in all groups treated with ISO, histopathological changes such as mononuclear cell infiltration, erythrocyte extravasation, myofibril loss, and intracytoplasmic vacuolization, as well as the HES, were increased compared with the control group (*p* < 0.05). The HES in the ISO-6-h group was increased compared with the control group (*p* < 0.05), similar in the ISO-24-h and ISO-3-day groups (*p* > 0.05), and higher compared with the ISO-7-day group (*p* < 0.05). In the ISO-24-h group, HES increased compared with the control group (*p* < 0.05), was similar to the ISO-6-h group (*p* > 0.05), and was higher compared with the ISO-3-day and ISO-7-day groups (*p* < 0.05). In the ISO-3-day group, HES increased compared with the control group (*p* < 0.05), was similar to the ISO-6-h group (*p* > 0.05), and decreased compared with the ISO-24-h group (*p* < 0.05). In the ISO-7-day group, HES increased compared with the control group (*p* < 0.05), while it decreased compared with the ISO-6-h, ISO-24-h, and ISO-3-day groups (*p* < 0.05) (Fig. 3).

### Levels of proinflammatory markers in cardiac tissue at different time points after ISO application

**Fig. 4:**
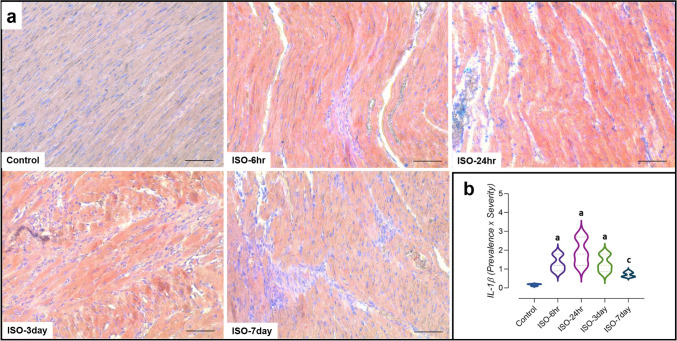
Cardiac IL-1β levels at different time points after ISO administration. Immunoreactivity of IL-1β in cardiac tissue increased at 6 h, 24 h, and 3 days after ISO administration compared with the control group. However, cardiac IL-1β levels decreased in the ISO-7-day group compared with the ISO-24-h group. **a** IL-1β IHC-staining microphotographs. Scale bar: 100 µm. **b** IL-1β IHC evaluation graph. ^a^Compared with the control group. ^c^Compared with the ISO-24-h group. *p* < 0.05. *IHC* immunohistochemical, *ISO* isoproterenol, *IL-1β* interleukin-1β

Cardiac IL-1β levels were increased 6 h, 24 h, and 3 days after ISO administration compared with the control group (*p* < 0.05). In the ISO-6-h group, cardiac IL-1β levels were increased compared with the control group (*p* < 0.05), and similar in the ISO-24-h, ISO-3-day, and ISO-7-day groups (*p* > 0.05). In the ISO-24-h group, cardiac IL-1β levels were increased compared with the control group (*p* < 0.05), similar to the ISO-6-h and ISO-3-day groups (*p* > 0.05), and higher compared with the ISO-7-day group (*p* < 0.05). In the ISO-3-day group, IL-1β levels were increased compared with the control group (*p* < 0.05), and similar to the ISO-6-h, ISO-24-h, and ISO-7-day groups (*p* > 0.05). In the ISO-7-day group, IL-1β levels were similar to the control, ISO-6-h, and ISO-3-day groups (*p* > 0.05), but decreased compared with the ISO-24-h group (*p* < 0.05) (Fig. 4).

**Fig. 5:**
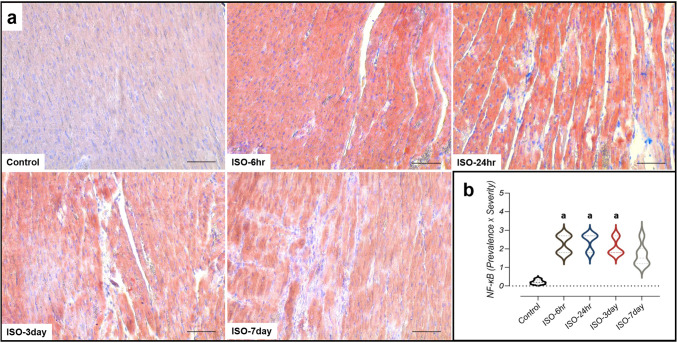
Cardiac NF-κB levels at different time points after ISO administration. Compared with the control group, NF-κB immunoreactivity in cardiac tissue increased 6 h, 24 h, and 3 days after ISO administration. However, cardiac NF-κB levels in the ISO-7-day group were not different from the control group. **a** Microphotographs of NF-κB IHC staining. Scale bar: 100 µm. **b** NF-κB IHC evaluation graph. ^a^Compared with control group. *p* < 0.05. *IHC* immunohistochemical, *ISO* isoproterenol, *NF-κB* nuclear factor kappa B

**Fig. 6:**
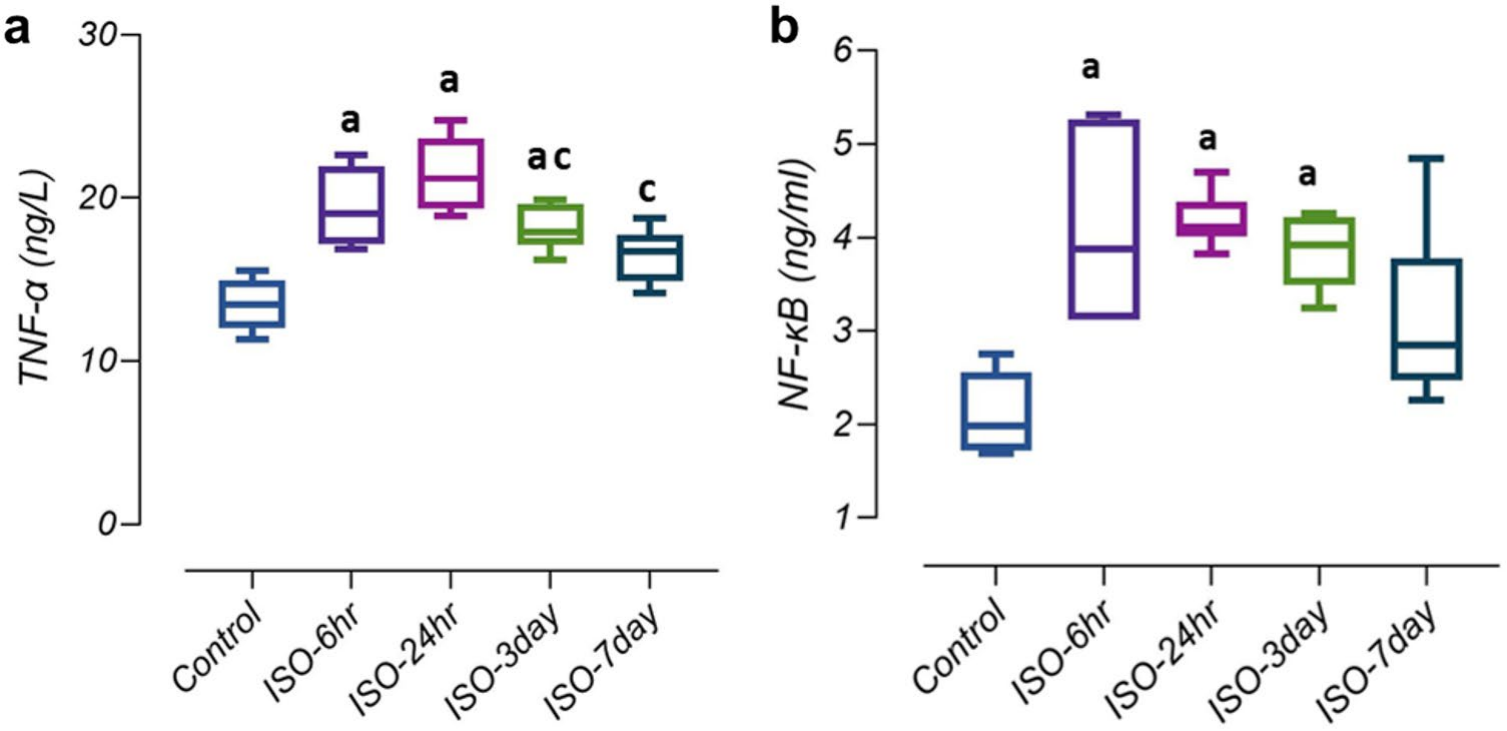
Cardiac TNF-α and NF-κB ELISA levels at different time points after ISO administration. TNF-α levels in cardiac tissue increased at 6 h, 24 h, and 3 days after ISO administration compared with the control group. However, TNF-α levels decreased in the ISO-3-day and ISO-7-day groups compared with the ISO-24-h group. NF-κB levels in cardiac tissue increased at 6 h, 24 h, and 3 days after ISO administration compared with the control group. However, NF-κB levels in the ISO-7-day group were not different from the control group. **a** Graph of cardiac TNF-α levels. **b** Graph of cardiac NF-κB levels. ^a^Compared with the control group, ^c^Compared with the ISO-24-h group, *p* < 0.05. *ISO* isoproterenol, *TNF-α* tumor necrosis factor-α, *NF-κB* nuclear factor kappa B

Cardiac NF-κB immunoreactivity and ELISA levels were increased 6 h, 24 h, and 3 days after ISO administration compared with the control group (*p* < 0.05). In the ISO-6-h group, cardiac NF-κB levels increased compared with the control group (*p* < 0.05), while they were similar to the ISO-24-h, ISO-3-day, and ISO-7-day groups (*p* > 0.05). In the ISO-24-h group, cardiac NF-κB levels increased compared with the control group (*p* < 0.05), while they were similar to the ISO-6-h, ISO-3-day, and ISO-7-day groups (*p* > 0.05). In the ISO-3-day group, NF-κB levels increased compared with the control group (*p* < 0.05), while they were similar to the ISO-6-h, ISO-24-h, and ISO-7-day groups (*p* > 0.05). ISO-7-day group NF-κB levels were similar to control, ISO-6-h, ISO-24-h and ISO-3-day groups (*p* > 0.05) (Figs. 5, 6).

Cardiac TNF-α levels were increased at 6 h, 24 h, and 3 days after ISO administration compared with the control group (*p* < 0.05). In the ISO-6-h group, cardiac TNF-α levels were increased compared with the control group (*p* < 0.05), but were similar to the ISO-24-h, ISO-3-day, and ISO-7-day groups (*p* > 0.05). In the ISO-24-h group, TNF-α levels were increased compared with the control group (*p* < 0.05), similar to the ISO-6-h group (*p* > 0.05), and higher than the ISO-3-day and ISO-7-day groups (*p* < 0.05). In the ISO-3-day group, TNF-α levels were increased compared with the control group (*p* < 0.05), similar to the ISO-6-h, ISO-3-day, and ISO-7-day groups (*p* > 0.05), but decreased compared with the ISO-24-h group (*p* < 0.05). In the ISO-7-day group, TNF-α levels were decreased compared with the ISO-24-h group (*p* < 0.05), but were similar to the control and other ISO groups (*p* > 0.05) (Fig. 6).

### OTULIN levels in cardiac tissue at different time points after ISO administration

**Fig. 7:**
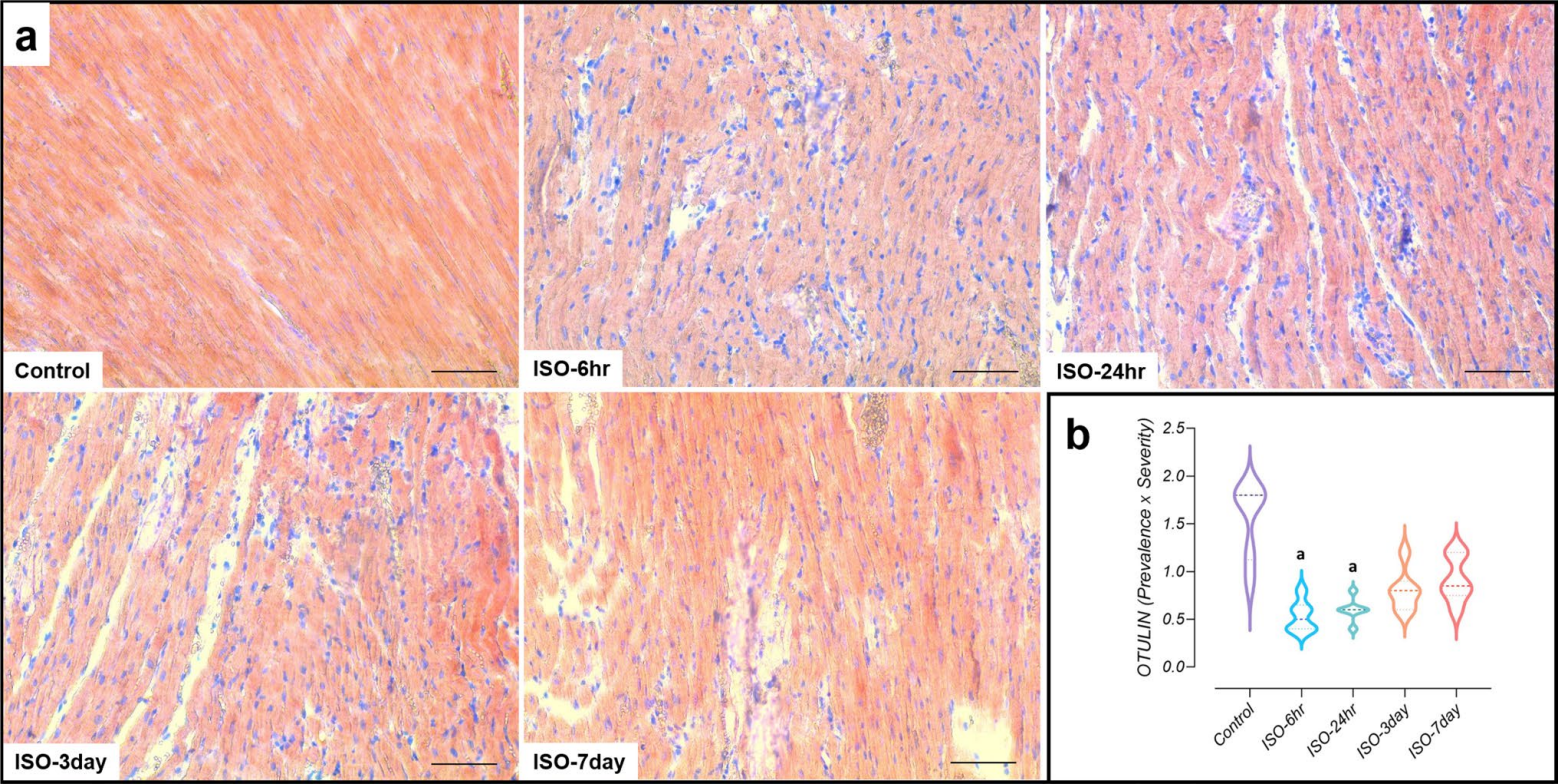
Cardiac OTULIN levels at different time points after ISO administration. Compared with the control group, OTULIN immunoreactivity in cardiac tissue decreased 6 h and 24 h after ISO administration. However, OTULIN immunoreactivity levels in cardiac tissue were not different from the control group in the ISO-3-day and ISO-7-day groups. **a** Microphotographs of OTULIN IHC staining. Scale bar: 100 µm.** b** OTULIN IHC evaluation graph. ^a^Compared with the control group. *p* < 0.05. *IHC* immunohistochemical, *ISO* isoproterenol

Immunoreactivity levels of OTULIN in cardiac tissue were decreased 6 and 24 h after ISO administration compared with the control group (*p* < 0.05). In the ISO-6-h group, cardiac OTULIN immunoreactivity levels were decreased compared with the control group (*p* < 0.05), but were similar to the other ISO groups (*p* > 0.05). In the ISO-24-h group, cardiac OTULIN immunoreactivity levels were decreased compared with the control group (*p* < 0.05), but were similar to the other ISO groups (*p* > 0.05). In the ISO-3-day and ISO-7-day groups, OTULIN immunoreactivity levels were similar to both the control and other ISO groups (*p* > 0.05) (Fig. 7).

**Fig. 8:**
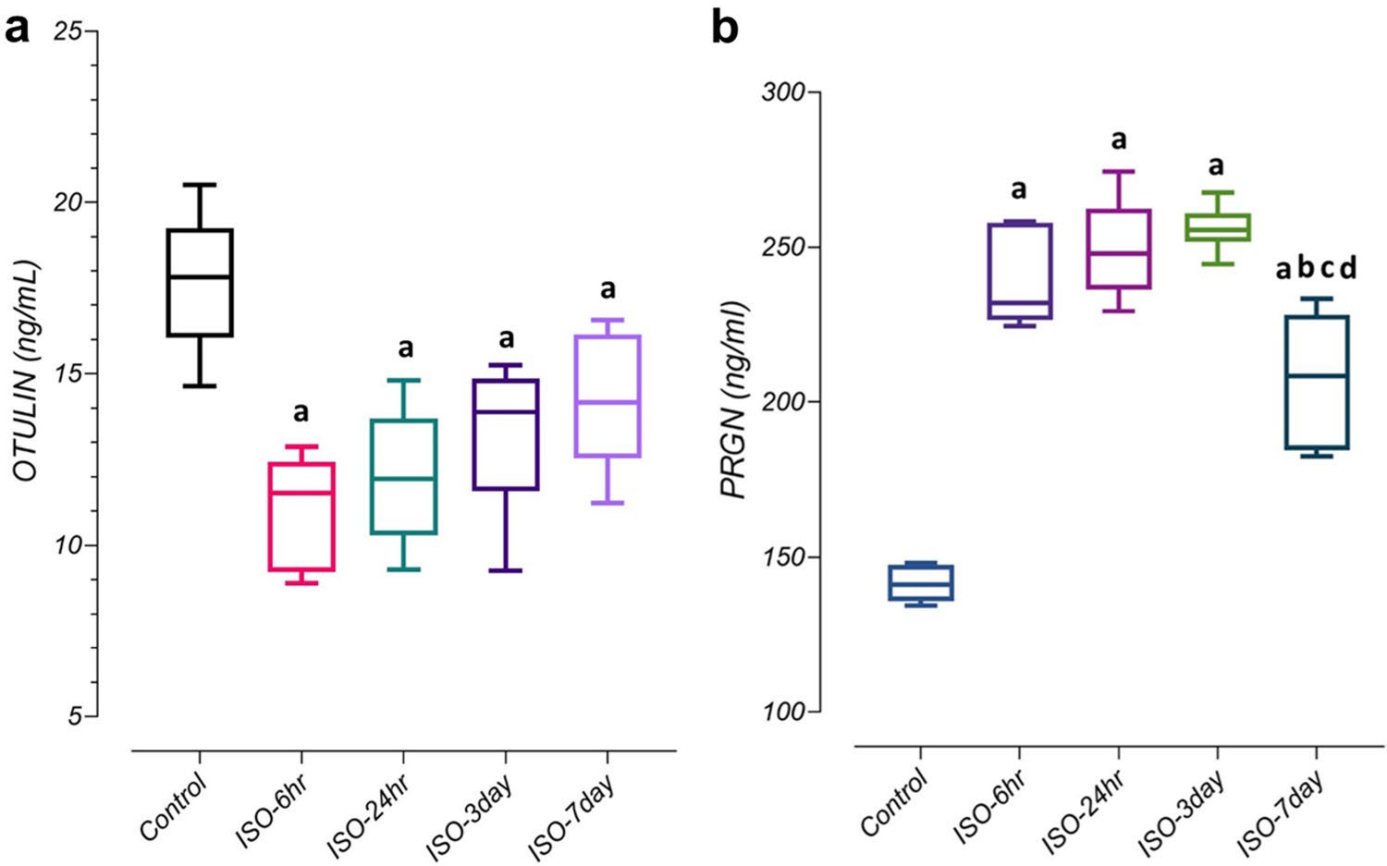
Cardiac OTULIN and PRGN ELISA levels at different time points after ISO administration. Cardiac tissue OTULIN ELISA levels decreased in all ISO-treated groups compared with the control group. Cardiac tissue PRGN ELISA levels increased in all ISO-treated groups compared with the control group. However, PRGN ELISA levels in the ISO-7-day group were decreased compared with all other ISO groups. **a** Graph of cardiac OTULIN ELISA levels. **b** Graph of cardiac PRGN ELISA levels. ^a^Compared with the control group. ^b^Compared with the ISO-6-h group. ^c^Compared with the ISO-24-h group. ^d^Compared with the ISO-3-day group. *p* < 0.05. *ISO* isoproterenol, *PRGN* progranulin

Similarly, OTULIN ELISA levels in cardiac tissue were decreased in all groups treated with ISO compared with the control group (*p* < 0.05). Heart tissue OTULIN levels were similar in the ISO-6-h, ISO-24-h, ISO-3-day, and ISO-7-day groups (*p* > 0.05). However, although a relative time-dependent increase in OTULIN levels was observed after ISO treatment compared with the control group, this increase was not statistically significant (*p* > 0.05) (Fig. 8).

### PRGN levels in cardiac tissue at different time points after ISO administration

Cardiac PRGN ELISA levels were increased at 6 h, 24 h, 3 days, and 7 days after ISO application compared with the control group (*p* < 0.05). In the ISO-6-h group, cardiac PRGN ELISA levels were increased compared with the control group (*p* < 0.05), similar to the ISO-24-h and ISO-3-day groups (*p* > 0.05), and higher than the ISO-7-day group (*p* < 0.05). In the ISO-24-h group, PRGN ELISA levels were increased compared with the control group (*p* < 0.05), similar to the ISO-6-h and ISO-3-day groups (*p* > 0.05), and higher than the ISO-7-day group (*p* < 0.05). In the ISO-3-day group, PRGN ELISA levels were increased compared with the control group (*p* < 0.05), similar to the ISO-6-h and ISO-24-h groups (*p* > 0.05), and higher than the ISO-7-day group (*p* < 0.05). PRGN ELISA levels were increased in the ISO-7-day group compared with the control group (*p* < 0.05), but decreased compared with the ISO-6-h, ISO-24-h and ISO-3-day groups (*p* < 0.05) (Fig. 8).

**Fig. 9:**
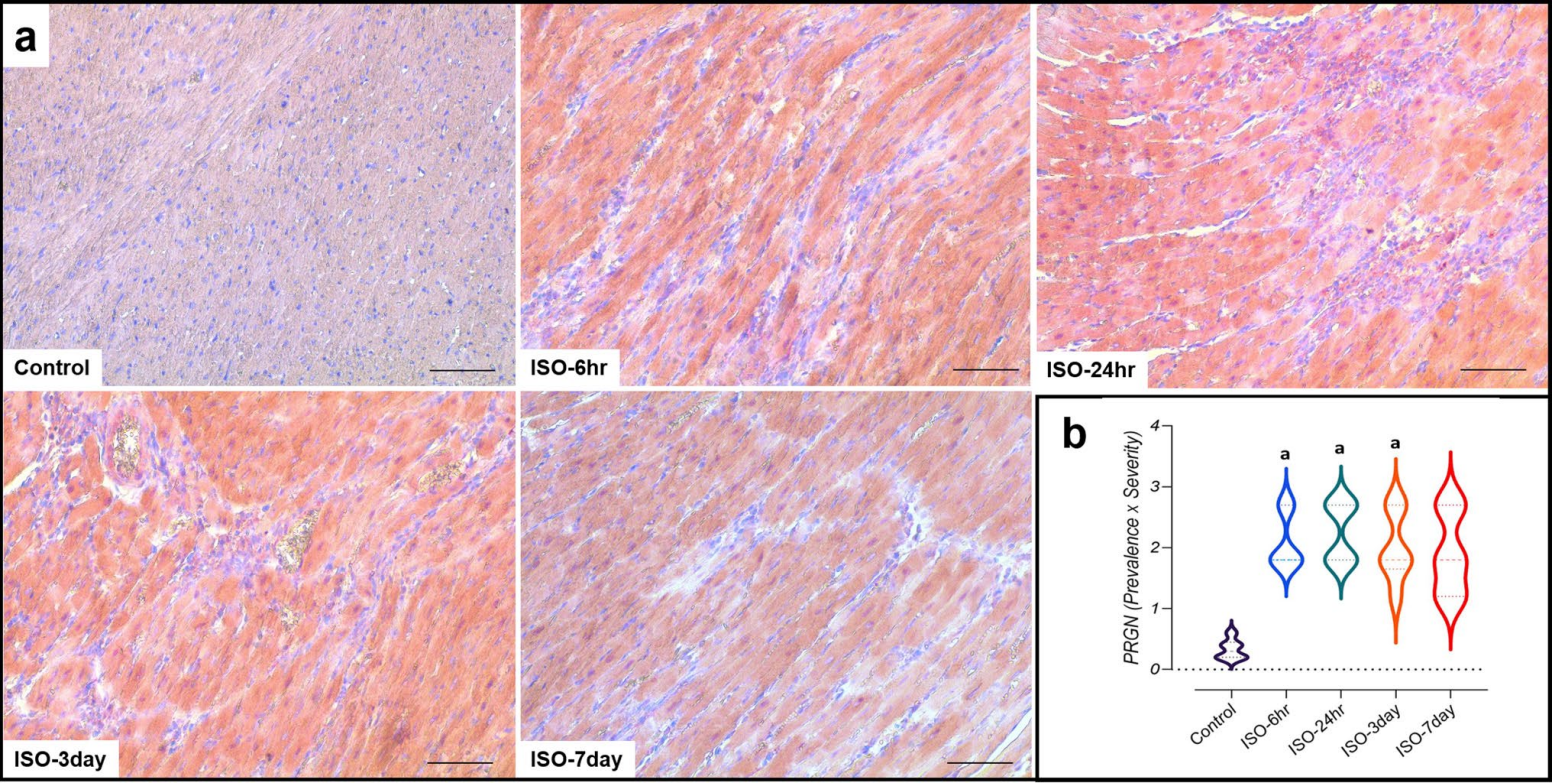
Cardiac PRGN levels at different time points after ISO administration. Compared with the control group, PRGN immunoreactivity in cardiac tissue was found to be increased at 6 h, 24 h, and 3 days after ISO administration. However, in the ISO-7-day group, PRGN immunoreactivity levels in cardiac tissue were not different from the control group. **a** Microphotographs of PRGN IHC staining. Scale bar: 100 µm. **b** PRGN IHC evaluation graph. ^a^Compared with the control group. *p* < 0.05. *PRGN* progranulin, *IHC* immunohistochemical, *ISO* isoproterenol

Similarly, cardiac PRGN immunoreactivity levels were increased at 6 h, 24 h, and 3 days after ISO administration compared with the control group (*p* < 0.05). In the ISO-6-h group, cardiac PRGN immunoreactivity levels were increased compared with the control group (*p* < 0.05), but were similar to the ISO-24-h, ISO-3-day, and ISO-7-day groups (*p* > 0.05). In the ISO-24-h group, cardiac PRGN immunoreactivity levels were increased compared with the control group (*p* < 0.05), but were similar to the ISO-6-h, ISO-3-day, and ISO-7-day groups (*p* > 0.05). In the ISO-3-day group, PRGN immunoreactivity levels were increased compared with the control group (*p* < 0.05), but were similar to the ISO-6-h, ISO-24-h, and ISO-7-day groups (*p* > 0.05). PRGN immunoreactivity levels in the ISO-7-day group were similar to those in the control, ISO-6-h, ISO-24-h, and ISO-3-day groups (*p* > 0.05) (Fig. 9).

## Discussion

This study revealed time-dependent changes in circulating cardiac marker levels and cardiac tissue histopathology during and after ISO-induced experimental MI and cardiac remodeling. Furthermore, the study determined the levels of oxidant/antioxidant parameters and proinflammatory cytokines in cardiac tissue at different time points during the MI process, based on oxidative stress and inflammation. We also present a report highlighting the potential role of PRGN and OTULIN in myocardial damage and cardiac remodeling.

During MI, changes in membrane permeability result in the release of certain markers, enzymes, proteins, and other molecules into the circulation (Singh et al. 2020). One study reported increased circulating CK-MB and Tn-I levels in an ISO-induced MI model (Yin et al. 2022). The release of the CK-MB enzyme, located in the myocardium and involved in muscle contraction, is associated not only with ischemia but also with myocytosis. Additionally, one of the most widely used biomarkers for the identification of myocardial injury is CK-MB. (Bostan et al. 2020). One study reported that not serial CK-MB values, but rather increased values over a longer period, were associated with subsequent cardiovascular events (Carvalho and Rassi 2016). However, troponin is a protein found in cardiac and skeletal muscle, but its I isoform has a very high specificity for the myocardium (Bostan et al. 2020). A study showed that an increase in troponin levels without an increase in CK-MB was associated with higher mortality (6.5% versus 12.5%), but in cases where CK-MB dynamics were present, an increase in troponin levels also increased mortality (6.8% versus 11.7%) (Yan et al. 2004). It has been reported that high levels of markers such as CK-MB in the myocardium can reflect changes in the degree of myocardial damage at different periods (Rao et al. 2023). Consistent with these results, this study found that circulating CK-MB and Tn-I levels increased at 6 h, 24 h, 3 days, and 7 days after ISO administration. However, it was determined that circulating CK-MB and Tn-I levels tended to decrease on the 7th day after ISO administration. In support of these results, it was observed that histopathological changes such as mononuclear cell infiltration, erythrocyte extravasation, myofibril loss, and intracytoplasmic vacuolization were commonly seen in cardiac tissue after ISO application, but similar to cardiac markers, histopathological changes decreased on days 3 and 7.

Lipid peroxidation is an endogenous chain reaction that results in the oxidative breakdown of lipids and the production of many oxidation products. Cardiomyocytes are negatively impacted in a number of ways by lipid peroxidation products. One important consequence of lipid peroxidation is MDA (Gianazza et al. 2019). Another lipid peroxidation product, 4-HNE, has been reported to affect biological functions, form protein adducts, and disrupt cellular homeostasis (Gao et al. 2024). Therefore, 4-HNE and MDA can be used as indicators of the severity of free radical-induced lipid damage (Kaya et al. 2025). In this study, increased levels of MDA and 4-HNE were detected in cardiac tissue after ISO application. However, CAT, a hemoprotein found in mitochondria and peroxisomes, plays a role in H_2_O_2_ removal (Pérez-Torres et al. 2019). Studies have reported decreased levels of antioxidant enzymes in cardiac tissue at the onset of experimental MI (rat model) (Shahzad et al. 2018; Rong et al. 2023). Similarly, in this present study, levels of the antioxidant enzymes GPx and CAT decreased in cardiac tissue after ISO administration, but increased significantly, particularly on day 7 after ISO administration. These results support studies suggesting that oxidative stress is a critical modulator of cardiac remodeling due to its importance and therapeutic potential in recent years (Shah et al. 2021; Martins et al. 2022).

Inflammation plays a significant role in MI development, with increased levels of proinflammatory cytokines (Kurian et al. 2016). TNF-α, the main inflammatory mediator, impairs myocardial contractility by stimulating the release of other cytokines and chemokines, including IL-1β. However, oxidative stress further damages cardiomyocytes, particularly when antioxidant defenses such as CAT are reduced (Mangali et al. 2019). One study showed that oxidative stress significantly contributed to MI progression with higher levels of proinflammatory markers such as cardiac IL-1β and TNF-α (Moris et al. 2017). Another study investigated inflammatory cytokine levels to compare different degrees of myocardial damage caused by 3-h, 6-h, and 24-h reperfusion following myocardial ischemia/reperfusion (I/R) injury in a mouse model. The study results reported that IL-1β and TNF-α levels increased at the onset of reperfusion but decreased afterward, although these inflammatory cytokine levels remained elevated for up to 24 h (Meng et al. 2023). In line with this information, this study observed an increase in cardiac proinflammatory cytokine (IL-1β, TNF-α, and NF-κB) levels after ISO administration. However, a significant decrease in cardiac proinflammatory cytokine levels was detected on day 7 after ISO administration. This may be related to the fact that induction of proinflammatory cytokines through stimulation of NF-κB cascades facilitates leukocyte traffic in the infarction, plays an important reparative role by clearing the infarction area of ​​dead cells, stimulating interstitial cell activation and angiogenesis. However, it is known that excessive and prolonged leukocyte activation can exacerbate damage, increase cardiomyocyte death, and stimulate protease-induced matrix degradation (Hilgendorf et al. 2024). When considered together, we believe that the decrease in NF-κB, IL-1β, and TNF-α levels on day 7 following ISO administration is related to positive cardiac remodeling.

Deubiquitinases regulate cellular metabolism and oxidative stress by acting on different substrate proteins. Furthermore, studies have shown that deubiquitinases, which play a regulatory role in heart disease, can control the initiation and progression of heart disease through a wide range of mechanisms (Zhan et al. 2024). OTULIN, a deubiquitinase, is considered to play a role in NF-κB-dependent inflammatory signaling and is an endogenous regulator necessary for controlling inflammatory responses (Yalcin et al. 2024a). One study reported that OTULIN inactivation destabilized TNF receptor-1 (TNFR1)-associated complex I, which is essential for NF-κB activation, and thus promoted the formation of cytoplasmic cell death signaling complexes (Heger et al. 2018). Furthermore, targeted deletion of OTULIN, which is expressed in peripheral immune cells, in these myeloid cells increased NF-κB activity, leading to inflammation and autoimmunity (Damgaard et al. 2016). A study investigating the time course of OTULIN expression in ischemic stroke in rats detected low levels of OTULIN and correlated this with sustained OTULIN expression in the brain. The same study reported that endogenous OTULIN expression rapidly and sustainedly increased in the cerebral cortex within 72 h after ischemic stroke, suggesting that OTULIN may be associated with resistance to ischemic brain damage (Xu et al. 2018). Similarly, this current study found that OTULIN levels decreased in cardiac tissue at 6- and 24-h time points after ISO administration, but showed an increasing trend on days 3 and 7 after ISO administration. We hypothesize that the dynamic changes in endogenous OTULIN levels in cardiac tissue during and after ISO-induced MI are related to proinflammatory cytokine levels and their role in cardiac remodeling.

According to recent research, PGRN protects the cardiovascular system by enhancing mitochondrial performance, modulating inflammatory pathways, and maintaining vascular integrity (Pogonowska et al. 2019; Zhou et al. 2019). Furthermore, PGRN plays a supportive role in cardiac repair after ischemic injury by reducing oxidative stress (Qiao et al. 2025). This also highlights the complex interaction between PGRN and cardiovascular homeostasis. Specifically, the potential of PGRN levels as a significant indicator of risk in patients with acute myocardial infarction (MI), both as a biomarker demonstrating disease severity and as a therapeutic target, has recently been emphasized (Sasaki et al. 2023). According to one study, PGRN concentration strongly linked with the severity of coronary artery disease, and serum PGRN levels were a significant risk factor in individuals with acute MI (Zhou et al. 2021). Similarly, this study also found that PRGN levels in cardiac tissue increased during and after experimental MI induced by ISO. However, although PRGN levels in cardiac tissue decreased on day 7 after ISO administration compared with 6 h, 24 h, and day 3, they remained high compared with the control group. According to a study on PGRN’s role in the pathophysiology of MI and I/R, PGRN deficiency worsens post-MI remodeling, which increases mortality, causes severe arrhythmias, and causes excessive left ventricular fibrosis (Sasaki et al. 2023). Interestingly, in these models, cardiac PGRN expression was reported to progressively increase on days 1, 3, and 7 after MI. This could be interpreted as an indication that PGRN-releasing neutrophils in myocardium that has suffered a myocardial infarction reduce myocardial damage by limiting further neutrophil migration and decreasing the inflammatory response (Sasaki et al. 2020).

Although valuable results were obtained in this study, its limitations include the lack of a gender distribution of the subjects and the failure to compare the trends of change at different time points between genders. In conclusion, investigating the cellular and molecular mechanisms involved in cardiac repair following MI is crucial for understanding the pathogenesis of post-infarction cardiac remodeling and heart failure. This study demonstrates that OTULIN and PRGN may be cellular effectors of cardiac repair and play a role in the molecular signaling of the restorative cardiac response. We believe that OTULIN and PRGN, which are associated with oxidative stress and inflammation, show promise as target molecules that can protect against MI and subsequent adverse cardiac remodeling.

## Data Availability

The datasets used and/or analyzed during the current study are available from the corresponding author upon reasonable request.
